# Compositional analysis in sorghum (*Sorghum bicolor*) NIR spectral techniques based on mean spectra from single seeds

**DOI:** 10.3389/fpls.2022.995328

**Published:** 2022-10-17

**Authors:** Gokhan Hacisalihoglu, Paul R. Armstrong, Princess Tiffany D. Mendoza, Bradford W. Seabourn

**Affiliations:** ^1^ Department of Biological Sciences, Florida A&M University, Tallahassee, FL, United States; ^2^ Stored Product Insects and Engineering Research Unit, United States Department of Agriculture-Agricultural Research Service (USDA-ARS) Center for Grain and Animal Health Research, Manhattan, KS, United States; ^3^ Department of Grain Science and Industry, Kansas State University, Manhattan, KS, United States

**Keywords:** sorghum protein, sustainable food systems, near infrared spectroscopy, seed content, oil, phenotyping

## Abstract

Sorghum (*Sorghum bicolor*) is an economically important cereal crop that can be used as human food, animal feed, and for industrial use such as bioenergy. In sorghum breeding programs, development of cultivars with desirable seed quality characteristics is important and development of rapid low-cost screening methods for seed nutritional traits are desired, since most standard methods are destructive, slow, and less environmentally friendly. This study investigates the feasibility of single kernel NIR spectroscopy (SKNIRS) for rapid determination of individual sorghum seed components. We developed successful multivariate prediction models based on partial least squares (PLS) regression for protein, oil, and weight in sorghum. The results showed that for sorghum protein content ranging from 8.92% to 18.7%, the model coefficients of determination obtained were $R_{CAL}^{2}=0.95$ (RMSEC= 0.44) and $R_{PRED}^{2}=0.87$ (RMSEP= 0.69). The model coefficients of determination for oil prediction were $R_{CAL}^{2}=0.92$ (RMSEC= 0.23) and $R_{PRED}^{2}=0.71$ (RMSEP= 0.41) for oil content ranging from 1.96% to 5.61%. For weight model coefficients of determination were $R_{CAL}^{2}=0.81$ (RMSEC= 0.007) and $R_{PRED}^{2}=0.63$ (RMSEP= 0.007) for seeds ranging from 4.40 mg to 77.0 mg. In conclusion, mean spectra SKNIRS can be used to rapidly determine protein, oil, and weight in intact single seeds of sorghum seeds and can provide a nondestructive and quick method for screening sorghum samples for these traits for sorghum breeding and industry use.

## Introduction

Sorghum (*Sorghum bicolor*) ranks fifth in world cereal production after corn, wheat, rice, and barley, with over 66 million tons produced (FAOSTAT, 2019). The United States is the leading sorghum producer (25% of world’s production and $2.4 billion in economic value) and Kansas is the leading state with half of the U.S. sorghum production (FAOSTAT, 2019; Hacisalihoglu and Armstrong, 2022). It is an economically important cereal and major grain crop for animal feed, and biofuels. Recent research on biofortification has focused on increasing seed nutrient value including protein, amino acids, beta carotene, and vitamins to make crops more nutritious without reducing crop yield (Hacisalihoglu et al., 2010; Murgia et al., 2013; Hacisalihoglu, 2020; Hacisalihoglu and Vallejos, 2005; Hacisalihoglu and Settles, 2017).

As a gluten-free source of protein, sorghum is increasingly used in human food, in antioxidant health promoting compounds and in snack foods. Therefore, measurement of seed nutritional quality traits and their variation are important as breeding parameters. (Ciampitti and Prasad, 2019). The current standard methods to quantify seed composition are costly, slow, destructive in nature, and sometimes not considered environmentally friendly. Near infrared spectroscopy (NIRS) coupled with multivariate calibration is an environmentally friendly, nondestructive, and fast method for seed nutrition quality analysis (Armstrong et al., 2011; Peiris et al. (2019)). More recently, single kernel NIR spectroscopy (SKNIRS) has been used in quality trait characterization of food crops such as corn, soybean, peas, and beans (Hacisalihoglu et al., 2010; Gustin et al., 2013; Hacisalihoglu et al., 2016; and Spielbauer et al. (2009); Hacisalihoglu et al., 2020). It has shown potential in variety identification, sorting based on protein content, and separating damaged from sound single wheat kernels (Delwiche, 1995; Delwiche, 1998; Peiris and Dowell, 2011). There also exists potential to identify double haploid seeds which can decrease cultivar development by several years as has been done in other crops such as maize in which haploid and hybrid seeds can be discriminated based on oil content (Hussain and Franks, 2019; Gustin et al., 2020).

It is highly useful for crop breeders and industry to measure single seed quality composition and subsequently select the desired traits easily and nondestructively. In this regard, NIR spectroscopy has significant advantages such as working with limited sample size, fast quantification without sample preparation and cost effectiveness (Armstrong, 2006).

It is challenging to develop SKNIRS methods for small-seeded crops such as sorghum and particularly for sorghum oil content which is small in concentration. Therefore, the objectives of the current study were: 1) to assess the feasibility of using SKNIRS for small-seeded crops; and 2) to evaluate calibration models to predict protein, oil, and weight in single intact sorghum seeds that could be used to indicate seed trait variability in samples as well as potentially sorting seeds by traits for breeders.

## Materials and methods

### Sorghum seed samples

**Figure 1:**
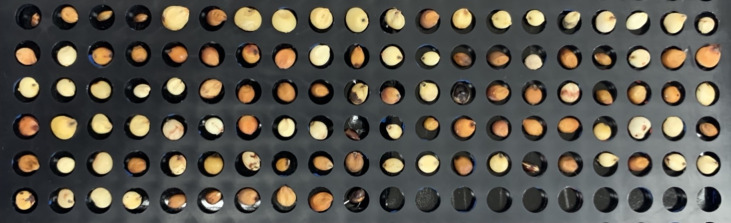
Sorghum accessions used for single NIR spectroscopy calibration prediction model development in the current study.

A collection of 108 widely diverse sorghum accessions (*Sorghum bicolor*) were selected and obtained from the U.S. Department of Agriculture (USDA) National Germplasm Center (https://npgsweb.ars-grin.gov/gringlobal). The samples were selected to maximize diversity based on size and country of origin, **Figure 1**.

### NIR spectroscopy measurements

NIR spectra were collected with a custom-built single-seed instrument. Armstrong (2006) provides a detailed description of the instrument and spectral data collection. Briefly, the instrument used was a 907-1689 nm InGaAs-based spectrometer (CD NIR-256-1.7T1, Control Development, South Bend, IN) where two fiber-optic cables were positioned at each end of an inclined, 8 mm diameter, glass tube and reflected light from seeds falling through the tube was collected. The glass tube was illuminated by 48 miniature halogen lamps along its length and circumference. Spectra were collected at 1 nm intervals and converted to absorbance values.

### Protein wet lab analysis and seed weight

Protein content (%, as-is) was measured from single whole seeds using a Leco FP-628 nitrogen combustion analyzer (St. Joseph, MI, USA) according to AACC Method 46-30.01 as described elsewhere (Hacisalihoglu et al., 2020). Individual seed weights were measured in triplicate with a balance. Total protein was calculated as N x 6.25.

### Oil analysis

Oil content (%, as-is) of the intact seeds was measured by a Bruker MiniSpec mq20 NMR Analyzer (Bruker Biospin, Billerica, MA, USA) as described previously (Hacisalihoglu et al., 2020). The oil content was obtained by placing 1 g of seeds into a 20 mm test tube warmed to 40°C, then inserting the tube in the NMR instrument using the standard procedures suggested by the manufacturer. The analyzer was calibrated prior to measurements and optimized for the range of oil to be measured. This small bulk sample was used as a reference for single seed oil as there is no practical way to measure oil content on such small seeds.

### Calibration model development and validation

Spectral data were analyzed using Unscrambler X software (CAMO Analytics, Oslo, Norway). Standard normal variate (SNV) was used as a pre-processing method for oil and protein. Partial least squares (PLS) regression was performed and evaluated using leave-one-out cross validation to select the optimal models. The samples were further subdivided into training (2/3 of total samples) and validation (1/3 of total samples) sets based on creating equal variability, as much as possible, in the biochemical seed parameter reference values. Model evaluation and selection were based on model factor levels as suggested by the Unscrambler software and evaluation of independent spectral data in the validation sets. For protein models, reference protein was the average protein from the three kernels from each accession. These values were indexed to the nine averaged spectra from the same kernels. For oil models, the nine averaged spectra were indexed to the oil content measured by NMR as previously described. Weight models indexed the three spectra average of a single seed to its true seed weight. **Table 1** summarizes the PLS modelling data.

**Table 1:** PLS modelling data.

	Single Seed Model Reference Spectra	Average Model Reference Spectra	Model Reference Trait
Protein	3 spectra averaged for each seed	9 spectra from 3 seeds averaged	Single seed-LECO
Oil	3 spectra averaged for each seed	9 spectra from 3 seeds avg	1 gm sample-NMR
Weight	3 spectra averaged for each seed	NA	Single seed-weight

## Results and discussion

### Mean spectra and single kernel NIR spectroscopy seed composition and weight parameters

**Figure 2:**
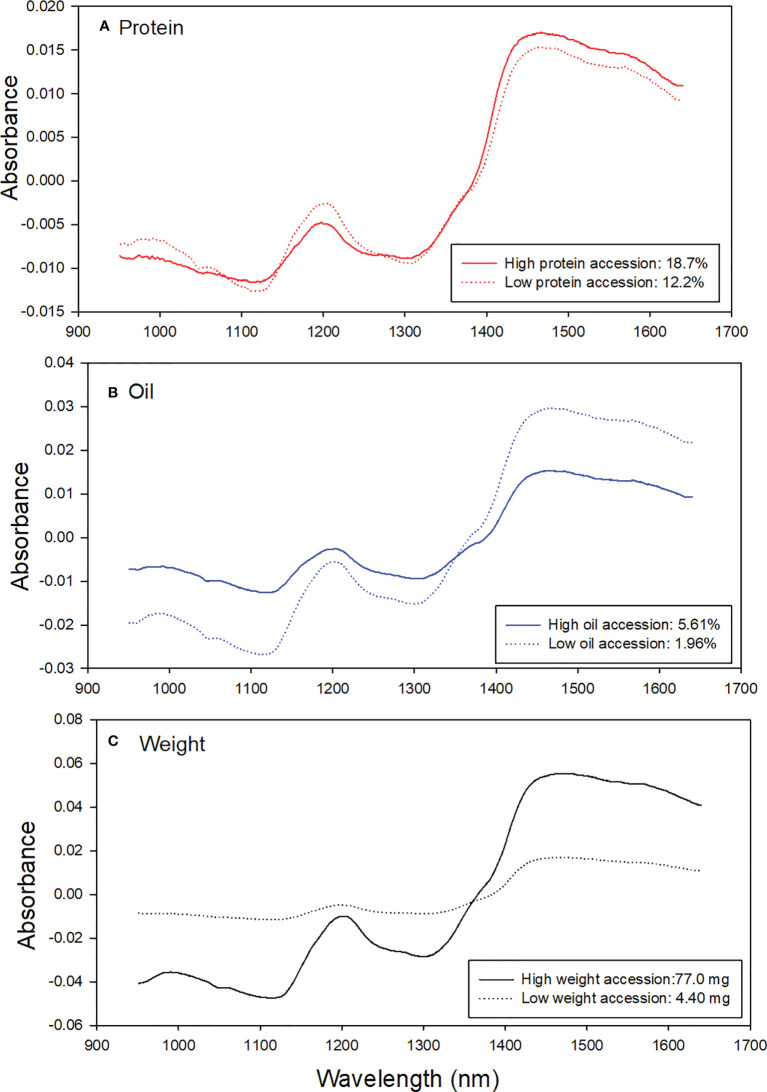
Frequency distribution for **(A)** protein, **(B)** Oil, and **(C)** weight for the sorghum accession.

Seed parameters of 108 diverse sorghum accessions represented by their means, standard deviations (SD), and ranges are summarized in **Table 2** and **Figure 2**. Analytical data from sorghum accessions showed a wide variation in protein content (8.92% - 18.7%), oil (1.96% - 5.61%), and weight (4.40 mg – 77.0 mg) (**Figure 2**). Furthermore, there were no statistically significant correlations detected between seed weight and protein or oil content (*p*< 0.01; **Table 3**). This is in agreement with Hacisalihoglu et al. (2020) who reported no strong relationship between protein and seed weight in peas.

**Table 2:** Reference protein, oil, and weight of 108 single sorghum seeds.

Parameter	Mean	SD	Range
Protein (%, as-is)	12.1	1.94	8.92-18.7
Oil (%, as-is)	3.23	0.79	1.96-5.61
Weight (mg)	28.8	13.5	4.40-77.0

**Table 3:** Phenotypic correlations between seed nutritional traits and weight in sorghum.

Traits	Protein	Oil
Oil	0.32	
Weight	-0.27	-0.13

**Figure 3:**
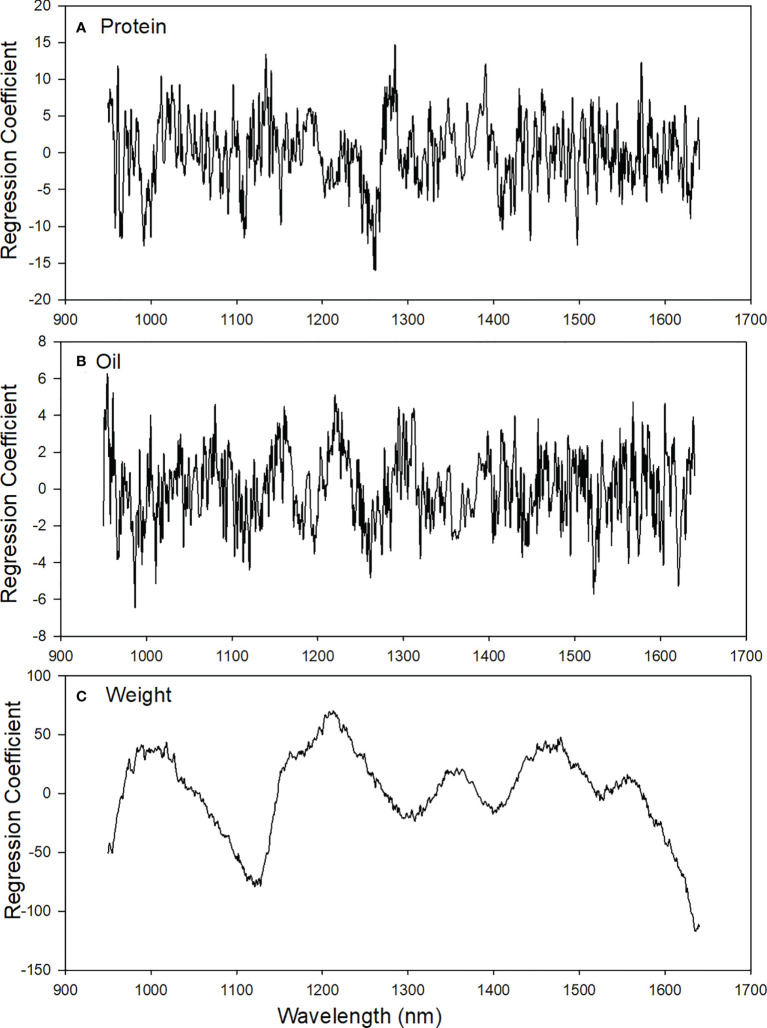
Examples of contrasting spectra of high- and low-contents of **(A)** protein, **(B)** Oil, and **(C)** weight in sorghum seeds.

**Figure 3** shows examples of contrasting spectra pairs of sorghum protein, oil, and weight. Each line represents average original SKNIR spectra of nine sorghum seeds. Absorbance differences were found between high- and low-protein sorghum accessions (**Figure 3A**), weight sorghum accessions (**Figure 3C**), and oil sorghum accessions (**Figure 3B**). The results show that average spectral absorbance differences between seeds with high or low composition and weight.

### Protein, oil, and weight calibration development

**Figure 4:**
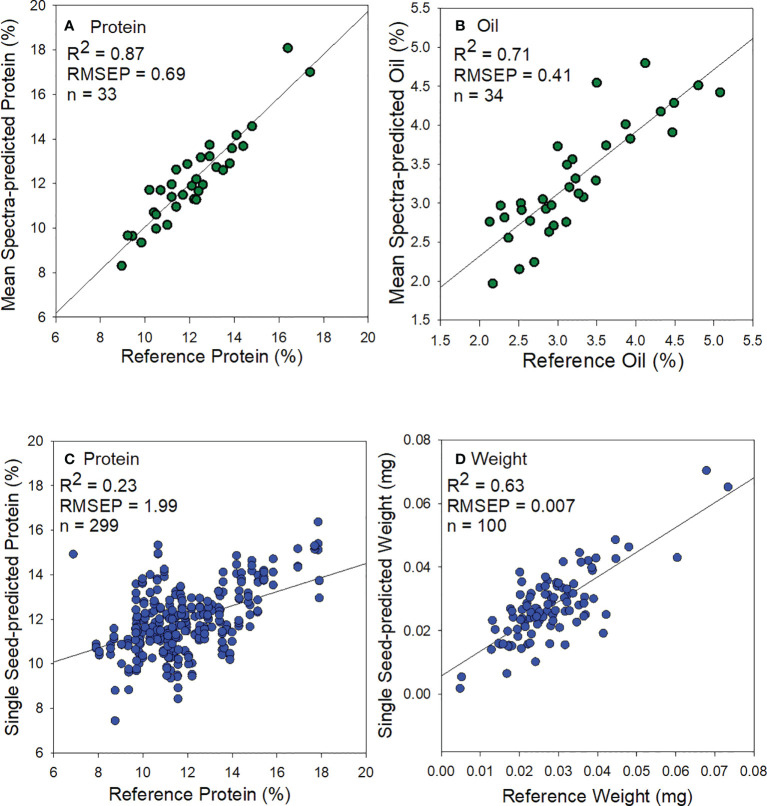
Scatter plots of predicted and analytically wet-lab measured **(A)** protein from using mean spectra, **(B)** oil from using mean spectra, **(C)** protein from single sorghum seeds, and **(D)** weight from single sorghum seeds.

The predicted *vs*. reference plots of external validation sets for protein, oil, and weight models are shown in **Figure 4** and model statistics **Table 4**. The PLS regression protein model using validation mean spectra showed good prediction accuracy (R^2 ^= 0.87, RMSEP= 0.69, **Figure 4A**). Similarly, the oil model showed reasonable accuracy for validation data (R^2 ^= 0.71, RMSEP= 0.41, **Figure 4B**) and would be appropriate for screening low or high oil sorghum samples. The weight model showed reasonable accuracy (R^2 ^= 0.63, RMSEP= 0.007, **Figure 4D**) but was lower than previous research on maize (Gustin et al., 2013) and common beans (Hacisalihoglu et al., 2010).

**Table 4:** Summary of calibration and prediction statistics for protein, oil, and weight models using mean and single seed spectra of sorghum.

CALIBRATION					PREDICTION		
Parameter	N	factors	RMSEC	R^2^	N	RMSEP	R^2^
**Average spectra**							
** Protein**	76	12	0.44	0.95	33	0.69	0.87
** Oil**	74	12	0.23	0.92	34	0.41	0.71
**Single seed**							
** Protein**	678	12	1.48	0.55	299	1.96	0.23
** Weight**	228	10	0.007	0.81	100	0.007	0.63

N is sample size; R^2^ is coefficient of determination; RMSEC is root mean standard error of calibration; and RMSEP is root mean standard error of prediction.

The protein model from single sorghum seeds showed poor prediction (R^2 ^= 0.23, RMSEP= 1.96, **Figure 4C**). Overall, mean spectral based models for weight determination in single intact sorghum seeds gave the best results for both calibration and prediction performances. Similar results have been reported with another small-seeded crop, namely chia seeds for bulk oil prediction (Serson et al., 2020).

### PLS regression coefficients and NIRS regions associated with sorghum traits

**Figure 5:**
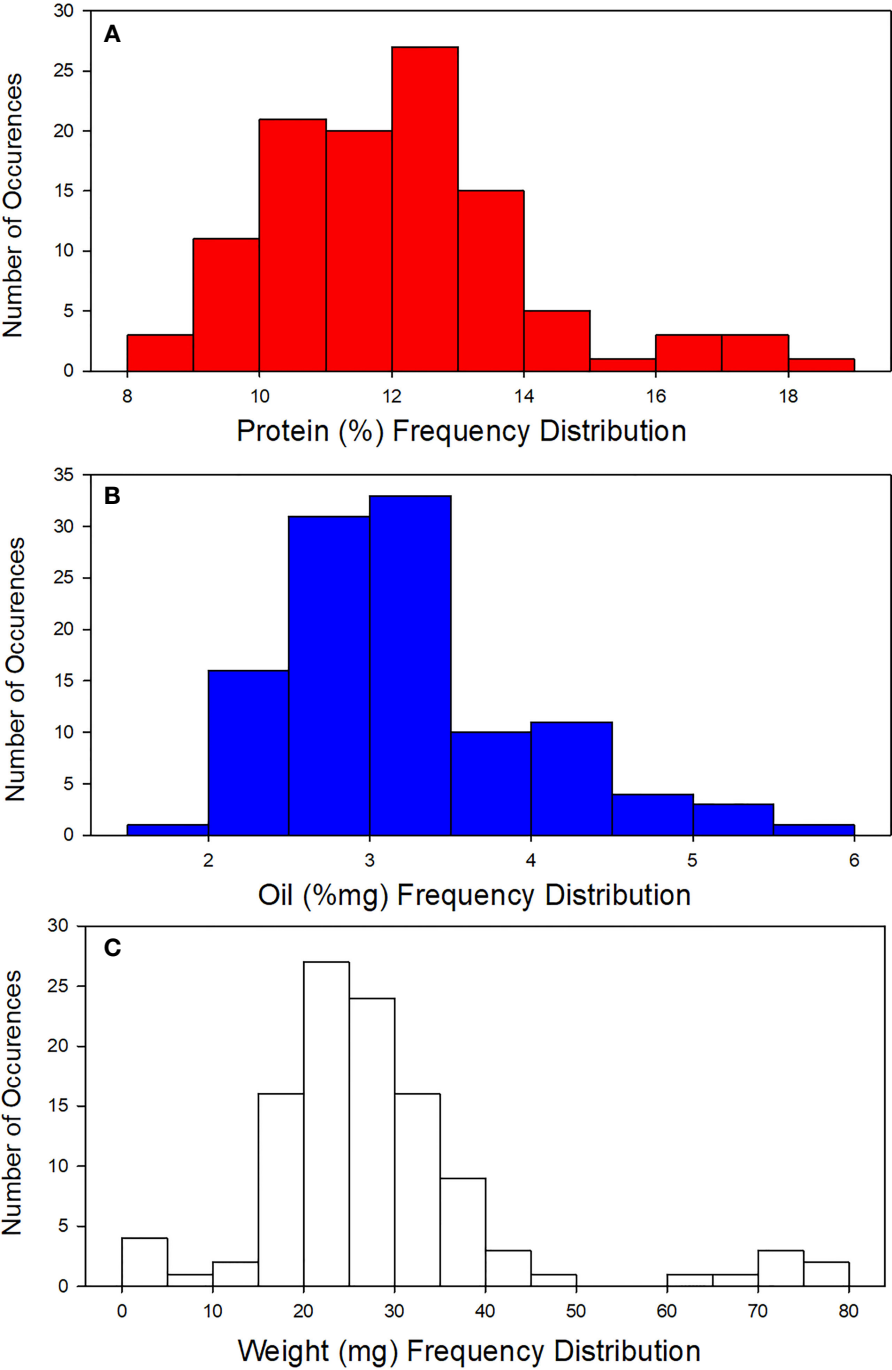
Beta-coefficient plots based on the optimal PLS models of **(A)** protein, **(B)** oil, and **(C)** weight in single sorghum seeds.

**Figure 5** shows regions of influence for PLS model coefficients. These regions are distinguished by large positive and negative coefficients which more heavily contribute to protein and oil prediction. The peaks for protein were observed for calibration models around 2^nd^ overtone N-H stretching (973-1020 nm; 1035-1040 nm; and 1498 nm). The peaks for oil were observed for calibration models around 3^rd^ overtone C-H stretching (992-1025 nm and 1210 nm) and 3^rd^ overtone O-H stretching (960 nm), **Table 5**. Prediction of weight cannot be explained in relationship to chemical composition and is thought to be more related to light quantity, i.e. large seeds may create different total reflectance over smaller seeds. As such better methods of modelling such as a summation of the absorbance spectra might provide better weight predictions over PLS modeling.

**Table 5:** Peak wavelength locations, their band assignment, and seed parameter associated with in single sorghum seed.

	Absorption	Parameter
Peak location	band assignment	associated with
992-1025 nm	C-H stretch of 3rd overtone	Oil related
1210 nm	C-H stretch of 2nd overtone	Oil related
973-1020 nm	N-H stretch of 2nd overtone	Protein related
1035-1040 nm	N-H stretch of 2nd overtone	Protein related
1498 nm	N-H stretch of 2nd overtone	Protein related
1123 nm	O-H stretch of 2nd overtone	Water related
960 nm	O-H stretch of 3rd overtone	Water, Oil related

## Summary and conclusions

To the best of our knowledge, this is the first study which has applied SKNIRS to predict sorghum seed protein, oil, and weight. Our findings in this paper are applicable to a broad range of sorting implementations in the field, industry, as well as sorghum breeding programs. The SKNIRS technique provides numerous advantages including: i) as wet chemistry methods of sorghum quality assessments are slow and costly, the SKNIRS method is a suitable, non-chemical alternative for classification or screening for protein, oil, and weight, nondestructively; ii) SKNIRS may provide a powerful, fast, high-throughput seed nutrient quality technique for sorghum breeding as well as uniformity screening activities; iii) SKNIRS can simultaneously quantify sorghum seed constituents; and iv) SKNIRS does not require sample preparation and therefore minimizes potential errors in the sample preparation process.

## Data availability statement

The raw data supporting the conclusions of this article will be made available by the authors, without undue reservation.

## Author contributions

GH developed the research hypothesis, designed the research methodology, performed the research, analyzed the data, and wrote the original draft with the contributions from PA, PM, and BS. PA developed the methodology and draft. PA and PM interpreted data analysis, helped with methodology and software. All authors contributed to the article and approved the submitted version.

## Funding

This study was supported by the United States Department of Agriculture (USDA-ARS) 1890 Research Sabbatical program to ARS Project 3020-43440-010-002-S and a subsequent Non-Assistance Cooperative Agreement with GH (FAMU).

## Acknowledgments

We thank USDA National Plant Germplasm Center (Griffin, GA) for providing the seeds. We thank Kevin Fay (Manhattan, KS) for his excellent technical help with nitrogen combustion. The mention of a proprietary product or trade name does not constitute a recommendation or endorsement by the US Department of Agriculture. The USDA is an equal opportunity provider and employer.

## Conflict of interest

The authors declare that the research was conducted in the absence of any commercial or financial relationships that could be construed as a potential conflict of interest.

## Publisher’s note

All claims expressed in this article are solely those of the authors and do not necessarily represent those of their affiliated organizations, or those of the publisher, the editors and the reviewers. Any product that may be evaluated in this article, or claim that may be made by its manufacturer, is not guaranteed or endorsed by the publisher.
